# “A whole ball of all-togetherness”: The interwoven experiences of intimate partner violence, brain injury, and mental health

**DOI:** 10.1371/journal.pone.0306599

**Published:** 2024-08-23

**Authors:** Danielle Toccalino, Halina (Lin) Haag, Emily Nalder, Vincy Chan, Amy Moore, Angela Colantonio, Christine M. Wickens

**Affiliations:** 1 Institute of Health Policy, Management and Evaluation, University of Toronto, Toronto, Ontario, Canada; 2 Acquired Brain Injury Research Lab, University of Toronto, Toronto, Ontario, Canada; 3 Lyle S. Hallman Faculty of Social Work, Wilfrid Laurier University, Waterloo, Ontario, Canada; 4 Temerty Faculty of Medicine University of Toronto, Department of Occupational Science and Occupational Therapy, Toronto, Ontario, Canada; 5 Temerty Faculty of Medicine, Rehabilitation Sciences Institute, University of Toronto, Toronto, Ontario, Canada; 6 KITE-Toronto Rehabilitation Institute, University Health Network, Toronto, Ontario, Canada; 7 Dalla Lana School of Public Health, University of Toronto, Toronto, Ontario, Canada; 8 Institute for Mental Health Policy Research, Centre for Addiction and Mental Health, Toronto, Ontario, Canada; 9 Campbell Family Mental Health Research Institute, Centre for Addiction and Mental Health, Toronto, Ontario, Canada; 10 Department of Pharmacology and Toxicology, University of Toronto, Toronto, Ontario, Canada; University of Michigan, UNITED STATES OF AMERICA

## Abstract

**Background:**

Intimate partner violence (IPV) is a global public health crisis, with physical violence leaving IPV survivors at high risk of brain injury (BI). Both BI and IPV have significant physical, psychological, cognitive, and social impacts, including a high risk of mental health concerns, yet there is limited exploration of IPV survivors’ experiences with BI and mental health. This study aimed to explore the BI- and mental health-related needs and experiences of IPV survivors from the perspectives of survivors and service providers with the objective of developing knowledge translation materials to raise awareness and support survivors and service providers in addressing these concerns.

**Methods:**

This qualitative interpretive description study involved 19 semi-structured interviews and two focus group discussions (2–3 participants each) with 24 participants including IPV survivors experiencing BI and mental health concerns as well as IPV, mental health, and BI service providers between October 2020 and February 2021. Three screening questions were used to identify probable BI among survivors. Participants across groups were an average of 48.5±12.7 years old and were predominantly cisgender women (96%), of European origin (75%), with a university degree (71%). Interviews were recorded, transcribed, and thematically analyzed.

**Findings:**

Across interviews, participants spoke about IPV, BI, and mental health as being complex and interrelated experiences that have impacts across the survivor’s life and extend well beyond the abusive relationship. Because of the underrecognized nature of BI in IPV, finding and accessing care requires persistence that survivors spoke of as being like “a full-time job.” The benefit of making meaningful connections, particularly with other survivors, was highlighted.

**Conclusions:**

Recognition of BI as a contributing factor shaping the lived experience of IPV survivors; acknowledgement that the impacts of IPV, BI, and mental health are far reaching and long lasting; and reducing barriers to finding and accessing appropriate care are critical to better supporting IPV survivors with BI and mental health concerns. Clinicians should consider BI and its lingering impacts among the IPV survivors with whom they work. Health and social policy that supports integration of care and the reduction of unnecessary barriers should be a priority.

## Introduction

Intimate partner violence (IPV)—physical, psychological, or sexual violence committed by a current or former spouse or other intimate partner [1]—is a pervasive public health concern globally. The most recent Statistics Canada report suggests that 44% of Canadian women have experienced IPV in their lifetime, more than half of whom have experienced physical violence [2]. Physical violence against an intimate partner is most commonly directed towards the head, face, and neck, including strangulation [3], a pattern of injury putting survivors at high risk of sustaining a brain injury (BI) [4,5]. BIs sustained by survivors can be categorized into traumatic brain injuries, referring to “an alteration in brain function, or other evidence of brain pathology, caused by an external force”[6], or hypoxic ischemic brain injuries, referring to a deprivation of oxygen and nutrients to the brain as a result of interrupted blood circulation [7,8]. Both types of BI can have significant and long-term cognitive, psychological, physical, and social consequences [9–12] that impact the individual, their families, and their support systems, regardless of the categorized severity of the injury (i.e., mild, moderate, severe) [13]. Despite the high rates of BI among IPV survivors identified in the literature, the significant personal and societal impact, and a growing awareness of the intersection of BI and IPV, the literature exploring this intersection is still quite young and IPV-related BI is often overlooked in policy and practice [4,14,15]. In general, there is a lack of both education and comfort or perceived competency around IPV among many service providers who do not specifically work in the IPV sector, particularly among healthcare professionals [16–18], despite provider knowledge being a recognized facilitator of help-seeking for IPV survivors [19]. Similarly, service providers supporting IPV survivors are generally underprepared to identify and support individuals with BI [20]. The complexities of BI add further challenge for service providers. Take rehabilitation professionals, for example. There is a recognized benefit of appropriate rehabilitation supports for individuals with BI to address neurological and cognitive challenges resulting from the injury [21,22]. Yet, a recent review looking at the role of rehabilitation professionals in IPV survivors’ care noted that only four studies considered BI, despite rehabilitation professionals being well equipped to support individuals with BI [17]. At the time of writing, we are aware of only a few programs in North America that provide specialized care for IPV-BI survivors [23–26], further emphasizing the need for broader awareness and action in both research and practice.

Independently, both IPV and BI are associated with increased occurrence and risk of various mental health concerns including but not limited to anxiety, depression, and post-traumatic stress disorder (PTSD) [27–32]. Rates and severity of mental health concerns–particularly anxiety, depression, and PTSD–are higher among IPV survivors with BI compared to those without [5] and BI symptoms can both overlap with and amplify symptoms of these three concerns [27,33–35]. This can make it difficult to distinguish between BI and mental health challenges in IPV survivors and lead to missed BI diagnoses [27,35]. Without proper identification of BI, IPV survivors are unlikely to receive appropriate healthcare for the BI [28,36], but may also experience ineffective mental health treatment as a result of the underlying BI going unaddressed [29,36]. Furthermore, survivors experiencing an unaddressed BI may have resultant neurological or cognitive challenges that may be interpreted as personal failure or disruptive behaviors rather than implications of a BI. These same challenges (e.g., challenges with memory, time management, emotional regulation, information processing) can also negatively impact a survivor’s capacity to make, attend, and actively participate in appointments. Despite the high rates of both BI and mental health concerns among survivors of IPV [4,29,30,37] and the high rates of mental health concerns among individuals with BI and vice versa [38–43], the literature investigating the intersection of BI and mental health among survivors of IPV is limited. Existing literature predominantly focuses on quantitative assessments of BI and prevalence of mental health concerns among IPV survivors [5]. While many articles focusing on the intersection of mental health and BI among IPV survivors noted implications for health and healthcare based on study findings, very few explicitly investigated healthcare use or experiences (e.g., [44,45]). Furthermore, these investigations have been predominantly conducted in the United States, a nation which relies largely on private insurance carriers, potentially limiting generalizability to publicly insured healthcare contexts such as Canada [5].

A better understanding of survivors’ experiences of IPV, BI, and mental health concerns, specifically as it relates to healthcare and healing, is required to better identify and address their needs. A recently published scoping review on this subject identified a need for investigation of survivor experiences to better inform recommendations and guidance for service providers, particularly those working in healthcare [5]. Qualitative approaches allow for a more fulsome exploration of survivor experiences, enabling a more informed, nuanced approach to related policy and practice [46,47]. To address the noted gap in exploration of survivor experiences and harness the benefits of qualitative approaches to explore lived experience, this research used qualitative interpretive description methodology [46,48] to explore the BI and mental health related needs and experiences of IPV survivors in Canada, particularly related to healthcare/healing from the perspectives of survivors and service providers.

## Methods

This qualitative interpretive description study was conducted as part of a multi-pronged project exploring the employment and mental health experiences of, and the impact of the COVID-19 pandemic on, women survivors of IPV with BI. The overarching research project aimed to address the occurrence of these events together, their combined impact on women survivors, and how to address the complex issues of stigma, safety, and support needs particularly in relation to mental health and employment contexts, which is currently overlooked in the literature [20,30,49]. In addition to contributing to a recognized knowledge gap, this research was conducted with the objective of developing knowledge translation materials to raise awareness of mental health concerns related to IPV and BI and to support survivors and organizations working with survivors in addressing these concerns. As the project had multiple foci (i.e., mental health, employment, and COVID-19) and discussions around these topics were rich, findings are reported across several manuscripts [50,51]. This manuscript specifically reports on the findings related to the research question: what are the BI- and mental health-related needs and experiences of IPV survivors, particularly related to healthcare or healing?

### Participants and sampling

Purposeful sampling was used to identify participants across four categories: women survivors of IPV; frontline workers; executive directors/program managers; and employers or human resources/union representatives. Though IPV impacts individuals across the gender spectrum, the majority of survivors are women (including cis- and trans-women), which informed our focus in this work on women-identifying survivors [52–54]. Participants were identified from a national Knowledge to Practice (K2P) Network consisting of over 300 diverse community partners, researchers, and survivors in the IPV and BI communities across Canada [20] and via snowball sampling. Individuals were eligible to participate if they were over 18 years of age; able to speak, read, and write in English; and able to provide informed consent. Survivors had to self-identify as women who have experienced IPV, executive directors/program managers had to be employed in a decision-making role in an organization providing services to IPV survivors, and frontline workers had to work in direct service provision for survivors of IPV or BI. As BI is so commonly overlooked in IPV survivors, we did not require formal or informal recognition of BI to participate in the study but instead asked three screening questions (1. “Have you ever experienced hits or injury to your head, face, or neck?” response options: yes/no; 2. “Did you lose consciousness, see stars, or feel dazed or confused during or after hits or injury to the head, face, or neck?” response options: yes/no; 3. “Did you and/or do you notice any ongoing challenges with any of the following after hits or injury to the head, face, or neck?” response options: list of challenges, check all that apply) adapted from established screening tools [55,56] in a brief demographic survey that also asked about formal healthcare services. All survivors interviewed endorsed experiencing hits or injury to the head, face, or neck and multiple ongoing challenges since experiencing those hits/injuries. Individuals who expressed interest in participating received a package including study information, a consent form, discussion questions, a demographic questionnaire, and a list of local resources for emotional support. Participants returned signed consent forms and contacted the study team via phone or email to schedule an interview. Survivors received a $100 gift card as an honorarium. Participants were recruited and interviewed between October 6, 2020, and February 19, 2021 and provided written informed consent before participating. Approval for this research was granted by the Research Ethics Boards at the University of Toronto (Protocol #39175) and Wilfrid Laurier University (Protocol #6611).

### Data collection & analysis

All participants completed a brief demographic survey prior to participating in interviews or focus groups. Semi-structured interviews and focus groups covered topics related to employment (e.g., experiences of employment as affected by IPV, desired support services for employment), mental health and healthcare (e.g., experiences of mental health, experiences and resources for healthcare and/or healing), and COVID-19 (e.g., changes to daily life, changes to support systems) via broad, open-ended questions to encourage discussion. Aligned with our research aims, participants were primed to think of the above topics in relation to their experiences with IPV and BI. Interviews and focus groups were co-facilitated by the first and second author, with the first author leading discussion related to mental health and healthcare. Both interviewers were cisgender White women who were pursuing doctorate degrees during this study, with training in facilitation and qualitative research methods, one of whom has a BI. Among the broader research team (all listed authors), a diversity of educational backgrounds, levels of training, and career stages were represented, ranging from masters’ level students to senior researchers across fields including social work, rehabilitation sciences, and health systems research. The first and second authors’ training in health systems and policy research and social work, respectively, informed the development of research questions and interview guides. These different perspectives allowed for exploration of survivor experiences in a way that could both inform changes to front-line practice as well as the need for broader health system change. Interviews lasted 60–90 minutes, were conducted via Zoom, audio recorded, and transcribed in full using a transcription service. Both interviewers took notes on the context and content of the session and participated in reflective memoing before and after each interview. Preliminary data analysis was an iterative process with data collection. Interviews were transcribed and transcripts reviewed by the first and second authors on a rolling basis to allow for the interview guide to be adjusted to further investigate topics of interest that arose as the project progressed. Preliminary analysis was conducted by the first, second, and fifth authors to identify discussion related to IPV-BI and the three categories of focus–mental health, employment, and COVID-19 –and develop preliminary coding. The three analysts met regularly throughout the process to discuss the codes being developed and their interpretation, contributing to the trustworthiness of the findings. Within the context of the full interview, portions of the discussion related to mental health and healthcare were analyzed by the first author using the six stages of reflexive thematic analysis as described by Braun and Clarke [57,58]. The analysis process began with refamiliarization with the data, including reading and/or listening to the entirety of all transcripts before focusing in on discussions related to mental health and healthcare. Focused portions of the discussion relevant to the research questions explored here were then coded by the first author and compared to the preliminary analysis completed on the full dataset. Codes related to mental health and healthcare were then grouped into potential themes, which were revised and refined in an iterative process with the broader research team as reports were being developed. Reporting in this manuscript was guided by Braun and Clarke’s proposed evaluation tool for thematic analysis [58]. Dependability and confirmability were established through an audit involving a methodologically self-critical account of research conduct [59]. Trustworthiness was established through triangulation of analysts, regular meetings to discuss codes and categories and their interpretation, and development of an audit trail.

## Findings

A total of 24 individuals across four categories (women survivors of IPV (n = 6); frontline workers (n = 7); executive directors/program managers (n = 5); and employers or human resources/ union representatives (n = 6)) participated in 19 interviews and two focus groups (one of frontline workers and one of employers with 3 and 2 participants, respectively). Based on the completed demographic forms, participants were, on average, 48.5±12.7 years old, predominantly identified their ethnicity as being of European origin (n = 18, 75%), and all but one (a cisgender man) identified as cisgender women. All but one participant had at least some post-secondary education, with most having a university degree (71%). Service providers had an average of 13.8±11.3 years of experience in the IPV sector working in organizations such as victim services, shelters, BI services, and advocacy. Most of the IPV organizations represented by service providers predominantly support cisgender women. All survivors endorsed experiencing at least one injury to the head, face, or neck as a result of IPV on the demographic form, and all but one (83%) endorsed losing consciousness or experiencing alterations in consciousness (e.g., feeling dazed or confused, seeing stars) as a result of that injury, indicative of a probable BI. Though not directly assessed on the demographics survey, several survivors also noted experiencing BI unrelated to IPV either before or after their IPV experience during their interviews. Survivors further endorsed numerous ongoing challenges commonly associated with BI, including headaches, memory problems, and trouble concentrating (Table 1).

**Table 1:** Challenges reported by survivors after experiencing injury to the head, face, or neck.

Challenges reported	Number of survivors reporting challenge (max 6)	%
Headaches	6	100%
Memory problems	6	100%
Fatigue or tiredness	6	100%
Trouble concentrating	6	100%
Difficulty multi-tasking	6	100%
Problems organizing tasks	6	100%
Sleep problems	5	83%
Flashbacks	5	83%
Depression or Anxiety	5	83%
Light or sound sensitivity	5	83%
Nightmares	5	83%
Dizziness	4	67%
Ringing in your ears	4	67%
Mood swings or anger	4	67%
Hard to follow conversations	4	67%
Loss of taste or smell	1	17%
Total Challenges (mean, SD)	13	1.67

We developed three major themes related to BI- and mental health-related needs and experiences of IPV survivors, particularly related to healthcare/healing: 1) experiencing mental health and BI as “a whole big ball of all-together-ness”, 2) experiences permeating into other aspects of life and beyond the relationship, and 3) finding and getting access to care as a “full time job.” Quotation material is embedded throughout the text identified by the group of participant (e.g., survivor, frontline worker) and their participant or focus group number (e.g., P1, FG1). We retained the participant’s use of terminology in quotation material (e.g., the use of ‘brain injury’ rather than the acronym ‘BI’) to maintain the participant voice in the reporting.

### Experiencing mental health and BI as “a whole big ball of all-togetherness”

Survivors and service providers spoke of a range of mental health challenges, with depression and anxiety being the most frequently discussed. A common thread across conversations was the inevitable melding of IPV, mental health, and BI experiences. Many survivors spoke of their mental health challenges as being life-long, with experiences both pre-dating and long outlasting their abusive relationship(s), making it challenging to determine whether it was a result of or exacerbated by their experience with IPV. Some survivors spoke about pre-existing mental health concerns being amplified by their experience of IPV.

*“I’ve always felt like a stress ball. I’ve always felt neurotic. I’ve always felt anxious … so, it’s hard for me to sort of figure out what came later, after the IPV stuff. But I know for sure like the sort of more imminent–is that the word?–like the sort of more immediate day and weeks and months following my issue, like certainly that was a whole other level.”–Survivor (P1)*

Others spoke about mental health concerns arising during or after their abuse. A survivor who had experienced numerous hits to the head, shared that “constant hitting in the head was his way of dealing with me I think because you don’t see it.” (Survivor P4) These injuries resulted not only in persistent migraines, but also had an impact on her mental health.

*“I ended up in depression really bad and I was hospitalized for it … there’s some of these issues [post-traumatic stress, depression, anxiety] that I never had in my life till then, after the fact. So, who’s to say, I might have ended up this way anyway, but I think it’s all the crap I went through and the stress–and it just depleted my whole body.”–Survivor (P4)*

This was echoed by service providers who recognized mental health concerns as very common among the survivors they support, with one manager (P8) sharing: “I think most of the women that we have worked with that have experienced IPV and BI also have another sort of significant mental health thing happening for them.” PTSD in particular was noted as being common among IPV-BI survivors, likely arising from the abuse:

*“But I’d say the vast majority of women are coming in with some sort of intrusive PTSD symptoms, nightmares, flashbacks, just really awful memories and they’re struggling with that and that’s how they view themselves as somehow “crazy”–that’s how they look at it.”–Frontline (P14)*

Though we asked about both mental health and substance use, the latter was discussed less frequently across both survivors and service providers. Increased substance use was discussed as a coping mechanism both during and after the abusive relationship.

*“I always smoked pot, I always drank, but never like I started to. So, it was only after, it was only after the IPV well, that crazy summer that I basically increased use of everything.”–Survivor (P1)*

Survivors also spoke about substance use being used as a mechanism of control by their abuser, that participating in whatever substance use their partner was involved in was one way to keep themselves safe. A survivor (P2) shared “If I didn’t [smoke] then it would be more violence.”

In addition to the mental health impacts of IPV, survivors spoke about the physical health impacts, including BI. Some survivors had received BI diagnoses. A survivor remembered “I was sort of just out of it and couldn’t really do much” (Survivor P1); she noted her doctor recommended cognitive rest “no watching TV, no reading books, no anything”, which was only possible for her because she could go stay with her dad instead of returning to her home with her abuser. Another survivor spoke to constraints in her personal and professional life, noting “there’s only certain things I could do with my brain injury” (Survivor P11). She further noted her BI impacted her ability to recognize her abusive relationship for what it was “I took many years of abuse and I didn’t realize it. I didn’t see it” (Survivor P11). Even though sometimes the likelihood of BI resulting from abuse seemed evident—one survivor shared “I was attacked with a tyre iron, and it was primarily my head that was attacked” (Survivor P5)—many survivors shared their difficulty realizing or accepting the physical impact that IPV had on them, particularly the physical injury to their brain. Predominantly, this was a result of not realizing that BI was possible or not recognizing what the impacts of BI might be.

*“Because I never considered I had a brain injury, I just figured it as the–you know, just the stress I was dealing with all the time. I put it down to that more than anything. And I think most women do that are in those situations.”–Survivor (P4)*

*“I never thought that my brain would be affected by any of the abuse issues*. *I thought*, *at least I can always think*. *And I couldn’t*.*”–Survivor (P5)*

This challenge was echoed by service providers, who spoke about the survivors they work with not making the connection between IPV and the impacts on their physical and mental wellbeing.

*“If you don’t know what’s wrong; if you’re not–you as a survivor–are not making the connection between the violence and the injury and the outcome in my abilities to function now. You can fall into this mire of despair and uncertainty and insecurity, and we know that all of those leads to or can lead to mental–impact on your mental wellness.”–Executive Director (P6)*

Conversely, when that connection between cognitive challenges and BI was made, survivors feel relief and validation.

*“And I think a little bit of a validation that they’re not alone in that. Often that is comforting. I haven’t actually met a woman that hasn’t become comforting when I’ve said, “There–we work with a lot of survivors that struggle with that same thing.” And they’re like, “Oh.” There’s like some validation that that is, that happens as a result of your head being injured.”–Program Coordinator (P10)*

IPV has a range of physical (e.g., BI) and mental health impacts that often meld together. Connecting a survivor’s lived experience to a possible cause, such as BI, can be validating and help them navigate their healing journey.

### “It doesn’t just end”: Experiences permeate into other aspects of life and continue beyond the relationship

Beyond the tangle of IPV, BI, and mental health experiences themselves, survivors spoke about the impact of these experiences on their lives more broadly, extending past the abusive relationship. For example, substance use that reduced risk of violence while in the relationship had longer-term implications, particularly when trying to leave the relationship.

*“Because I did use [cannabis] when I was with him and how that impacts you when you leave—and when you have a child because then you’re going through family court, it’s another way that there can be that sense of power and control, because, you know, it’s just another mechanism that they can use against you.”–Survivor (P2)*

Survivors also noted challenges with executive function resulting from the BI that, for many, persist to this day.

*“Dealing with the stress and the physical and mental impact—it’s hard to function … And it does impact your ability to think and to coordinate and to organize and to multitask. I find now I can’t multitask very well at all.”–Survivor (P4)*

This lasting challenge with organization and multitasking, a common impact of BI, was noted by service providers as a gendered societal expectation of IPV survivors, who are predominantly women and many of whom are also parents. They noted that men who have experienced BI are typically not held to those same standards. Service providers spoke about expectations for women, both from the women themselves and from societal messaging more broadly, to multitask and function at a higher capacity than men with BI are expected to, despite the ability to multitask being negatively impacted by their BI. This includes childcare responsibilities, which often fall on women, but extends to other aspects of personal and work responsibilities.

*“There’s still an expectation that they are able to function at a higher capacity. Like that they can multitask and raise the kids and run the home and go to work and schedule everything. It is–there are so many multitasking expectations, I think, on women. And that is the thing that all the women that we’re working in IPV/BI say they struggle with “I cannot multitask” … Women are also great at masking that and often appearing like they can but are totally struggling with it behind the scenes. So, I think that is one of the big challenges I’m seeing, is, yea, the expectation that she can manage it all.” -Coordinator (P10)*

In addition to the cognitive effort of multitasking, service providers also noted that individuals with family responsibilities, which are more likely to fall on women, have less time to dedicate to healing from and adapting to life with a BI, which can impact their healing journey.

*“They are on all the time, because as a parent … [you] need to multitask all the time. There’s no downtime. The fatigue for women around that and again for any woman is tough, let alone someone who is dealing with, you know brain fatigue and brain fog constantly … Many of our male clients live alone. We know that the challenges around the success rate of marriage and relationships after a brain injury aren’t good … and so, they have a lot of time to meditate, mindfulness, relax, nap … there is a different ability for them to structure their time than I’m seeing with our women.”—Coordinator (P10)*

Finally, survivors emphasized that there is no end date after which the impact will no longer be felt.

*“It doesn’t just end. I mean, it doesn’t just end in terms of like the impact of the brain injury, but also doesn’t end in terms of the impact on–like just the trauma, right? … I wish that I could say that I, that there’ll be a time that I would be the same as I was prior to. I’m never going to be.”–Survivor (P2)*

#### Shame and stigma: “Why did I survive this near-death experience to be scum?”

Intertwined with the interrelated experiences of mental and physical health and the extension of the experience beyond the abusive relationship were experiences related to a survivors’ sense of worth and purpose. Survivors spoke about the compounding and interrelated experience of IPV, BI, and mental health concerns impacting how they see themselves, resulting in or contributing to a lack of self-worth.

*“I think with the mental health side of it and the IPV together, it melts together. When you have just the IPV or the TBI [traumatic brain injury] you lose a lot of yourself and then you lose a lot of self-confidence and self-esteem because you realize what you’ve lost … so is like a cumulative effect and you just keep getting pushed down and pushed down and pushed down till you feel like you’re less than the scum on the bottom of someone’s shoe. And you’re like, why did I survive this near-death experience to be scum? Like why? What is my purpose here?”–Survivor (P11)*

Survivors and service providers further spoke about shame and stigma around both experiences of mental health and BI. With mental health, participants across categories spoke about the stigma of being considered “crazy” and the impact that has on care seeking.

*“A lot of people, not just women, a lot of people don’t want to go on a drug or they don’t want to even see a psychiatrist, because the stigma, if you see a psychiatrist you’re crazy. And they don’t want anybody to know they have mental health issues.”–Survivor (P4)*

Discussions around BI similarly reflected shame and stigma, particularly around the concept of the brain being “damaged.”

*“I think, for whatever reason, there’s a lot of shame around sort of the concept that your brain is damaged … women are so frequently told how stupid they are. And, you know, this is such a tool of coercive control, that I just–it’s just something that I think women are–have a really hard time with.”–Manager (P8)*

Service providers also recognized that survivors’ feelings around worthiness were impacted by external messaging coming from their abuser and society, particularly around what it means to be a ‘good’ parent or a ‘productive’ member of society, which contribute to feelings of shame.

*“So, I think, you know, women do hear all the time, you know, you’re stupid, you could never support the kids on your own. Like you don’t know what you’re doing, you can’t get a job … Most women are hearing messaging about themselves that’s intending to undermine their sense of confidence and their sense of independence.”–Manager (P8)*

*“I think*, *also*, *for survivors of brain injury*, *that there’s a tremendous amount of shame about not being a productive member of society and many times these survivors have been quote/unquote “productive” members of society*. *They’ve been employed or fulltime or*, *you know*, *kind of self-sufficient and then this injury happens … I notice that shame is one of the biggest inhibitors to any kind of recovery*, *both for brain injury and intimate partner violence*.*”–Manager (P8)*

Survivors live with the implications of IPV, including BI and mental health concerns, across all aspects of their lives and well beyond their abusive relationship. Recognizing and validating that the impact “doesn’t just end” and the effects on survivors’ sense of worth and purpose can support survivors in their healing journey and “recalibrating to the new reality” (Manager P8).

### Finding and getting access to appropriate care, “it’s a full-time job”

Based on our demographic survey, all survivors interviewed endorsed receiving formal healthcare services for something related to IPV in their lifetime. Through our discussions, survivors spoke about seeking support both during and after leaving their abusive relationships from a variety of healthcare professionals including family doctors, medical specialists, emergency department personnel, rehabilitation professionals such as physiotherapists and occupational therapists, mental healthcare providers, BI support workers, acupuncturists, and massage therapists. The journeys to and experiences with care were quite variable across survivors, but several unifying themes were salient across experiences.

Survivors expressed their frustration with getting service providers to take them seriously and to recognize the severity and impact of their physical and emotional abuse. The most salient example shared involved the survivor being bounced from provider to provider, experiencing significant delays in care, losing their primary care provider, and having a probable BI entirely overlooked.

*“He smashed my head up against the wall over a dozen times … and when I phoned my family doctor she said, just go to emerge … and I had actually finally gone to emerge, and they said just go to your family doctor. And I said I want a scan. And ‘no, we don’t do that, I’m sure you’re fine. Here do you need some antidepressants, do you need some sleeping pills?’ Sorry, that’s not helping. It took me two months to see my family doctor, knowing what happened to me, two months. And all she did was dismiss it … I stood up to her and I said, you are not providing me healthcare service, you’re putting me in danger. And she says, well if you don’t like it here, you don’t have to come back. And they removed me from their service.”—Survivor (P13)*

Experiences like these were, unfortunately, quite common. Service providers noted challenges with getting survivors to access the care they might need because of past negative experiences with or perceptions of healthcare due in part to racism and stigma around substance use and poverty.

*“You kind of want them to be assessed at the hospital, or at least be looked at, and women are refusing, right? That’s not what they want to do. Women’s experiences, especially if there is substance use, are not positive at the hospital or with many services. And so, they’re not looking to have any more intrusive examinations or contacts with other people in that moment … to my experience, no one has ever really accepted transportation to the hospital.”–Executive Director (P3)*

*“So*, *from clients I have spoken to throughout my career*, *there’s definitely identified racism in our community*, *as there is*, *I would say in probably all*. *Certainly folks*, *they don’t want to go to the hospital because of that racism experience*. *So yeah*, *I think that’s probably the biggest barrier there is*.*”–Executive Director (P3)*

Aligned with the challenge of accessing care is the challenge of care not being available to support certain groups, in part due to a lack of funding.

*“The skin you’re in, the way you move through the world impacts your abilities to access resources, because again who gets funded? It isn’t Black women, it isn’t trans women, it isn’t Indigenous women, it isn’t usually women living with disabilities. So, the how depends on the who, who are you providing the resources for.–Executive Director (P6)*

Experiences with and perceived usefulness of services was quite individual to the survivor and the care provider. After the initial hurdle of finding and getting access to services that appreciate the implications of IPV, including BI and mental health, survivors spoke about the additional hurdle of finding services that worked for their unique situation. For example, massage therapy was a major component of the healing journey of one survivor while being triggering for another.

*“There are a lot of these things [health services] that are helpful, but women don’t know about them, because it’s not–it’s sort of trial and error, what works for some person, might not work for another person”–Survivor (P4)*

Knowing what supports to look for and how to find them was a skill survivors emphasized as being critical to getting appropriate care. Many credited their own professional experiences as key factors in their own knowledge of supports and success in finding them.

*“Because I worked in the field–in the behavior therapy field for over 15 years, and I have a brain in my head and I can be tenacious at times, I had the wherewithal to just keep going. To keep looking on the internet and phoning people and digging up–digging up what I could, as far as helping myself. So, the internet provided a lot more assistance than any human being did for the longest time. I don’t know what–women who do not have that background; I don’t know how they stay sane or even alive.”–Survivor (P13)*

Survivors also emphasized the importance of being persistent in seeking and gaining access to those supports, recognizing that those who do not know or have the capacity to be persistent will likely fall through the cracks.

*“I wish someone had just told me, like in life, you’re just going to have to be persistent and follow-up on everything all the time. Like, that’s just you know, like it’s a full-time job it feels like sometimes to follow-up on things, but that’s just it. So, not taking no for an answer, questioning things, challenging things, asking why. Asking for rationale and trying to gain an understanding.”–Survivor (P1)*

Finally, survivors spoke of the importance and benefit of making meaningful connections through the process. They spoke of the benefit of having service providers with whom they had built relationships and trust over time. One survivor shared that a trusted specialist she had been seeing for decades was able to get through to her about the danger of her situation when her friends and family had not.

*“I guess my biggest, the biggest change for me as an individual was a specialist. I had a specialist who actually said, ‘If you go back to your abuser, you’re signing your death certificate.’ And that’s when everything clicked in my head … My family was telling me, my friends were telling me, everyone was telling me, but it wasn’t till I saw the specialist and he just, I don’t know, he meant it”–Survivor (P11)*

Aligned with our earlier theme of finding meaning, all the survivors we spoke with emphasized the importance of supporting other survivors, citing both the emotional value for themselves and the empowerment of others when paying it forward.

*“So, I thought, well if I can make it better–the route, the paths, whatever a woman has to take to get the services that she needs and make the services a little better for her, then all that shit I went through for 20 years will be worth–you know, something good will have come of it.”–Survivor (P4)*

*“I want every woman who experienced what I experienced to have the same opportunity and be given the same strength—because I had to borrow strength—to be given the same strength to be the best that they can*.*”–Survivor (P5)*

While each survivor’s care journey looks different, challenges finding and getting access to care were ubiquitous, with many survivors relying on their own professional experiences to navigate the healthcare system. Trusting relationships with service providers and other survivors were meaningful supports on their healing journeys.

## Discussion

This interpretive description study set out to explore the BI- and mental health-related healthcare needs and experiences of IPV survivors through both survivor and service provider perspectives.

As the title quotation suggests, the experience of IPV, BI, and mental health meld together into a complex and interrelated experience that can be challenging to unravel, with BI being a particularly overlooked aspect of that experience. The pervasiveness of mental health concerns and BI among IPV survivors noted by both survivors and service providers in this work aligns with previous research [5,25] as does the exacerbation and persistence of mental health challenges following IPV-related BI [45]. This research is the first, to our knowledge, providing qualitative exploration of survivor experiences of these three overlapping and intertwined experiences. Findings from this research study focusing on care provision, which are reported in detail in a forthcoming manuscript [51], highlight the importance of recognizing BI as a thread in the ‘ball of all-togetherness’ and how critical recognizing the components and having a flexible approach to care are to providing survivors with appropriate care [27–29,36]. Without knowing what is wrong, survivors feel “broken” or “crazy” and service providers see survivors as defiant, difficult, or treatment resistant. Consequently, the act of identifying and naming the lived experience of BI was noted as validating for survivors, in addition to being critical to finding them appropriate care.

The concept of IPV, BI, and mental health as a ‘ball of all-togetherness’ also speaks to the complexity of these experiences both individually and when they interact. BI, IPV, and mental health challenges are each complex issues that are dynamic and unique, requiring a varying array of support based on the individual’s needs. For example, while there are many treatment and support avenues that may be useful, no two BIs are exactly alike. Finding a combination of supports that works is a very individual experience, particularly when mental health and substance use challenges also need to be addressed [60–63]. As such, there is a strong, recognized need for integrated, person-centred care for BI and mental health that is currently systemically unaddressed [64]. The need for an integrated and personalized approach is even more important for IPV survivors with BI and mental health concerns, as survivors emphasized. Survivors spoke about finding appropriate care as being a ‘full-time job’–identifying services, navigating barriers to access, and the ‘trial and error’ of finding a good fit were all challenges they encountered. Integrated people-centred health services, as described by the World Health Organization’s (WHO) Framework, would address these issues, emphasizing a need for a continuum of care coordinated across and beyond the health sector to meet individuals’ needs across the life course [65]. The WHO Framework calls out the current healthcare model’s focus on “disease-based and self-contained ‘silo’” as “undermin[ing] the ability of health systems to provide universal, equitable, high-quality and financially sustainable care.” [pg.1 [65]] A Canadian summit addressing priorities related to healthcare and support services for survivors of IPV with BI identified integrated care and “breaking down silos” as a top priority to better support survivors [66].

Social and societal factors also contribute to the complexity of IPV, BI, and mental health. One poignant example is the role of gender norms in perceptions of BI, IPV, and parenting, as discussed by service providers in our study. The responsibility of parenting often falls on women, particularly when parents separate, as often occurs following BI and in cases of IPV. As discussed by our service providers, when a relationship breaks down after a man experiences a BI, they rarely take on the bulk of parenting responsibilities. This allows them space to pursue activities and rest that is supportive of their healing. Women survivors of IPV with BI are often not provided that space, with the demands of parenting potentially impacting healing and health concerns (both BI and mental health related), which in turn can impact with their parenting [67,68]. In addition, women survivors of IPV with BI often have additional responsibilities related to leaving the relationship (e.g., navigating family courts, finding housing) acting as further barriers to their healing. Complicating this experience are the gendered impacts of BI. Women generally report worse outcomes after BI than men, particularly regarding mental health and prolonged post-concussion symptoms, which can also negatively impact their healing and longer-term outcomes [69,70]. Notably, negative long-term impacts can occur after any severity of BI [13], with some studies suggesting worse outcomes after mild to moderate injury [12,71]. The convergence of these experiences further emphasizes the need for social and health care sectors that are sensitive and responsive to the complexity of the experience of IPV survivors with BI, including the role of gender.

### Strengths, limitations, and future work

This research, to our knowledge, is the first qualitative exploration of healthcare experiences of IPV survivors with BI and mental health concerns, allowing for a more in-depth understanding of these intertwined experiences. It is also the first to integrate both provider and survivor perspectives, and the first to explore healthcare experiences among IPV-BI survivors in a publicly funded healthcare system (i.e., Canada). This contribution to the growing knowledge base around IPV-BI expands the understanding of survivors’ lived experiences and their healthcare needs from multiple perspectives (i.e., survivor and service provider) and in previously un- or under-explored contexts. It is our hope that this work encourages further exploration of this topic. In line with our research objectives, a mental health module was developed from these findings and is freely available on the *Abused and Brain Injured Toolkit* (www.abitoolkit.ca). As the overarching study of which this was part had a three-part focus–mental health, employment, and COVID-19 –interviews covered all three topics which was novel. Overall, this research further contributes to the understudied field of BI and mental health among women survivors of IPV.

Despite the valuable novel contributions of this work, there are several limitations to this research, which we discuss below alongside recommendations for future work that would address the limitations and/or contribute to notable gaps in the literature. Firstly, BI among survivors was assessed using several screening questions. While this is the standard for identifying BI in vulnerable populations, including IPV survivors [56], this approach does not allow for examination of findings by injury severity. While the three-part focus of the research enabled more insight into how mental health, employment, and COVID-19 interacted with each other, the conversation might not have the same depth as if the entire interview was focused solely on mental health. This work was conducted early in the pandemic, requiring a pivot from the initially planned, in-person interviews to a virtual modality. This shift may have impacted who was willing and able to participate, particularly among survivors. While all of the survivors interviewed had left their abusive relationships before the pandemic started and spoke of experiences prior to the pandemic’s impact, they also spoke of their ongoing experiences living with BI and mental health challenges as being exacerbated by COVID-19, which is further discussed elsewhere [50]. The pandemic had many impacts on IPV survivors, and an emerging body of literature has focused on these impacts [14,50,72–74]. However, further exploration into the lasting impacts, both positive and negative, and what implications the pandemic is having for IPV-BI survivors, their mental health, and their care is needed.

All participants were cisgender and service providers predominantly supported cisgender individuals. More research engaging with trans and gender diverse communities is needed to explore the complexity of their experiences and ensure that social and health care supports are designed in such a way as to support all survivors. We also recognize that a focus on women survivors necessarily excluded men and may exclude transfeminine or non-binary folks who do not identify as women. There is generally a paucity of research investigating IPV-BI across the gender spectrum that would benefit from exploration moving forward [5]. Similarly, most of our participants identified ethnically as being of European origin, though many service providers interviewed support racially and ethnically diverse clientele, so the nuances of the experience of racialized or ethnic minority survivors deserves further exploration. Finally, all but one of our participants had at least some post-secondary education, the impacts of this and other social determinants of health, such as income or geographic location, were not explored here and should be considered in future research.

### Implications & conclusions

This research contributes an in-depth discussion of the experiences of IPV, BI, and mental health as interrelated concerns from the perspectives of both survivors living with this interwovenness and service providers trying to untangle the threads. Without knowing about BI and, consequently, not knowing what to look for, survivors and service providers alike are unaware of what the implications could be. Recognizing BI as a contributor to the lived experience of IPV survivors; acknowledging that the impacts of IPV, BI, and mental health extend beyond the abusive relationship; and reducing the barriers to finding and accessing appropriate care, such as through integrated care models, are critical to better supporting IPV survivors with BI and mental health concerns.
